# GPS2 Is Required for the Association of NS5A with VAP-A and Hepatitis C Virus Replication

**DOI:** 10.1371/journal.pone.0078195

**Published:** 2013-11-04

**Authors:** Guodong Xu, Xiu Xin, Congyi Zheng

**Affiliations:** 1 State Key Laboratory of Virology, College of Life Sciences, Wuhan University, Wuhan, China; 2 China Center for Type Culture Collection, Wuhan University, Wuhan, China; Rosalind Franklin University of Medicine and Science, United States of America

## Abstract

Hepatitis C virus (HCV) nonstructural protein 5A (NS5A) is a component of the replication complex associated with various cellular proteins. It has been reported that G protein pathway suppressor 2 (GPS2) is a potential NS5A-binding factor, as identified in a yeast two-hybrid screens of human cDNA library using viral proteins as baits [1]. In this study, we demonstrated the interaction between GPS2 and NS5A in mammalian cells by coimmunoprecipitation analysis and found that both exogenously and endogenously expressed GPS2 interacted with NS5A of genotype 1b and 2a. Mutagenesis study demonstrated that Domain I of NS5A and coiled-coil domain of GPS2 are responsible for the interaction. Knockdown of GPS2 in hepatoma cell lines suppressed the replication of HCV RNA, which can be rescued by the expression of an RNAi-resistant GPS2. Furthermore, overexpression of GPS2 enhanced the association of NS5A with a proviral cellular factor, human vesicle-associated membrane protein-associated protein A (VAP-A), while knockdown of GPS2 disrupted interaction between VAP-A and NS5A. Taken together, our results suggest that GPS2 acts as a bridge between NS5A and VAP-A and is required for efficient HCV replication.

## Introduction

As a growing public health concern, Hepatitis C virus (HCV) infects about 170 million people worldwide and frequently leads to chronic hepatitis, liver cirrhosis and hepatocellular carcinoma (HCC) [2]. HCV is a single-stranded and positive sense RNA virus within the family *Flaviviridae*. Its genome encodes a single precursor polyprotein composed of about 3,000 amino acids (aa), which is cleaved by both cellular and viral proteases to generate three structural (core, E1, and E2) and seven nonstructural (p7, NS2, NS3, NS4A, NS4B, NS5A and NS5B) proteins [3]. Current treatment options for chronic hepatitis C are limited and accompanied with side effects [4]. Thus, there is an urgent need to develop effective therapeutic strategies against this human pathogen.

NS5A is an essential component of the HCV replicase and also strictly required for HCV assembly, but little is known about its precise function or mechanism [5]–[8]. NS5A exists as multiple phospho-isoforms and contains three discrete domains following the N-terminal membrane-anchoring α-helix [9], [10]. While no known enzymatic function has been ascribed to NS5A, it interacts with a wide variety of host cell proteins, such as FK506-binding protein 8 (FKBP8) and vesicle-associated membrane protein-associated protein A (VAP-A), and exerts a wide range of effects on cellular pathways and processes, including innate immunity, host cell growth and proliferation [11]–[13]. VAP-A, also called VAP-33, is an integral membrane protein with a coiled-coil domain and is widely expressed in human tissues [14]. Previous studies have shown that VAP-A binds to HCV NS5A and NS5B proteins and is important for the assembly of the HCV replication complex [13], [15]. Another VAP family member, VAP-B has also been reported to be involved in HCV replication through interaction with NS5A and NS5B [16].

Recently, G protein pathway suppressor 2 (GPS2) has been identified to interact with HCV NS5A in a high-throughput yeast two-hybrid (Y2H) screens of human cDNA library using viral proteins as baits [1]. GPS2 is a ubiquitous protein that was originally identified while screening for suppressors of Ras activation in the yeast pheromone response pathway [17]. Several studies have indicated the role of GPS2 in transcriptional regulation through interaction with a series of transcriptional regulators, such as the NCoR/SMRT nuclear receptor corepressor complex [18]–[20]. However, a critical cytoplasmic role for GPS2 in inhibiting the proinflammatory TNF-α pathway is also unveiled [21].

Here we first demonstrated the interaction between GPS2 and NS5A using co-immunoprecipitation analysis. Mutagenesis analysis revealed that domain I of NS5A and N-terminal coiled-coil domain of GPS2 are responsible for their interaction. Using retroviral-based RNA interference assay, we demonstrated that GPS2 is required for HCV replication. In addition, we found that GPS2 is involved in the efficient association between NS5A and VAP-A, which is critical for the formation of HCV replication complex and HCV RNA replication.

## Materials and Methods

### Cell Lines

Huh7, Huh7.5.1, Huh7 Lunet cells (kindly provided by professor Xinwen Chen, Wuhan Institute of Virology) and HEK293T cells were maintained in monolayer culture in Dulbecco’s modified minimal essential medium (DMEM; Hyclone) supplemented with 10% FBS, 100 U penicillin ml^−1^ and 100 U streptomycin ml^−1^ in a humidified atmosphere at 37°C with 5% CO_2_. Con1 cells harboring the subgenomic HCV replicon (kindly provided by professor Xinwen Chen, Wuhan Institute of Virology) were maintained in the complete DMEM supplemented with 0.5 mg G418 (Merk) ml^−1^ [22].

### Plasmids and Reagents

Mammalian expression plasmids for GPS2, VAP-A, NS5A, GPS2 and NS5A truncation mutants were constructed in a cytomegalovirus promoter-based vector containing a 5′-HA or -Flag tag by standard molecular biology techniques. Anti-FLAG, anti-HA, anti-actin mAbs, mouse IgG and Polybrene were purchased from Sigma Aldrich. Anti-core mAb were obtained from Santa Cruz Biotechnology. Mouse polyclonal GPS2 antiserum was raised against bacterially expressed and purified His-tagged human GPS2 N-terminus (aa 1–105) according to standard procedures. Anti-NS5A (9E10) was kindly provided by professor Charles Rice (Rockefeller University, New York, NY) [23].

### Retroviral Particle Production and Huh7 and Huh7.5.1 Cell Transduction

Double strand oligonucleotides corresponding to the target sequences were cloned into the pSuper.Retro.Puro plasmid (oligoengine). Vesicular stomatitis virus glycoprotein (VSV-G)-pseudotyped retroviral particles were produced in 293T cells by co-transfection of pSuper.Retro.Puro encoding short hairpin RNA (shRNA) with vectors encoding compatible packaging proteins pGag/Pol and VSV-G. Forty-eight hours post-transfection, cell supernatants were collected and cleared with a 0.45 µm filter, and used to transduce Huh7 cells and Huh7.5.1 cells at a multiplicity of infection (MOI) of 3 with Polybrene (10 µg/ml) overnight. The day after, fresh medium containing puromycin (2 µg/ml) was applied to select retrovirus-infected cells. Stable knockdown cell lines were routinely maintained in medium containing puromycin (2 µg/ml). The following sequences in GPS2 mRNA (GenBank accession number: NM_004489) were targeted: sh-GPS2 1# (GAACAGAAGATGAAGGAAG), sh-GPS2 2# (GATTATATCTTCAACACCA), sh-GPS2 3# (CCATCGTGGGTGAGGGTAT).

### RNA Extraction and Quantification of HCV RNA by Real-time RT-PCR

Total RNA from cultured cells and HCV RNA in culture supernatants were extracted using TRIzol and TRIzol LS reagent (Invitrogen) respectively according the manufacturer’s instructions. First-strand cDNA was synthesized from 1 µg of total RNA in a 20 µl total volume by using a recombinant reverse transcriptase (Roche) with random primers. Real-time reactions were carried out using a CFX96 real-time PCR detection system (Bio-Rad) with SYBR Green under the following conditions: 3 min at 95°C, followed by 40 cycles of 95°C for 30 s, 58°C for 30 s, and 68°C for 30 s. Sixty one cycles of 10 s, with 0.5°C temperature increments from 65 to 95°C, were used for the melting curves. Relative intracellular HCV RNA levels are the quantity of HCV RNA normalized to GAPDH RNA levels. Relative cellular gene RNA levels are the quantity of the specific gene RNA normalized to GAPDH RNA levels. HCV RNA levels in culture supernatants were determined relative to a standard curve comprised of serial dilutions of plasmid containing the HCV JFH-1 cDNA. The primer pairs used for HCV, GPS2, GAPDH are 5′-TCTGCGGAACCGGTGAGTA-3′ and 5′-TCAGGCAGTACCACAAGGC-3′, 5′-GAGAAGCACCAGCTTTTCCTGC-3′ and 5′- GAACAGTCAGGCTCTGCTGGTA-3′ and 5′-CCACTCCTCCACCTTTGAC-3′ and 5′-ACCCTGTTGCTGTAGCCA-3′, respectively.

### Immunofluorescence Analysis

Culture supernatant from GPS2 knockdown Huh7.5.1 cell lines and control cells infected with JFH1 for 72 h were collected to inoculate naïve Huh7.5.1 cells plated in 96-well plate. Three days later, cells were washed twice with PBS, fixed with 4% paraformaldehyde-containing PBS for 20 min at room temperature, and then permeabilized in 0.3% Triton X-100-containing PBS for 15 min. Blocking was performed in PBS with 10% FBS, 3% BSA and 0.3% Triton X-100 for 1 h at room temperature. Following three rapid washes, cells were labeled with mouse anti-core (Santa Cruz Biotechnology) primary antibodies diluted in 3% BSA, 0.3% Triton X-100–PBS for 1 h. Cells were washed three times in PBS and then labeled with FITC conjugated secondary antibodies diluted in 3% BSA, 0.3% Triton X-100–PBS for 1 h. Cells were extensively washed and incubated with DAPI for 10 min and extensively washed. Then cells were examined by laser-scanning microscopy (Olympus).Con1 cells transfected with HA-GPS2 were fixed with 4 % paraformaldehyde and permeabilized with 0.3 % Triton X-100 in PBS. After blocking, samples were incubated with NS5A mAb (9E10; 1 : 400) and HA Rabbit mAb (Cell Signaling Technology, 1∶500) for 1 h, then washed and incubated at room temperature for 1 h with Alexa Fluor 488-conjugated anti-mouse secondary antibody (1 : 500) and Alexa Fluor 555-conjugated anti-rabbit secondary antibody (1 : 500) (Invitrogen). Cells were extensively washed and then examined by laser-scanning microscopy (Olympus).

### Co-immunoprecipitation and Western Blotting

For co-immunoprecipitation in Huh7 or HEK293T cells, cells were seeded in 10 cm dishes 24 h before transfection. The plasmids were transfected by calcium phosphate precipitation in HEK293T cells and Lipofectamine 2000 (Invitrogen) in Huh7 cells, respectively. Twenty-four hours after transfection, the cells were washed with ice-cold PBS and solubilized with 1 ml lysis buffer (15 mM Tris, 120 mM NaCl, 2 mM EDTA, 0.5% Triton X-100, 10 µg/ml aprotinin, 10 µg/ml leupeptin, and 0.5 mM phenylmethylsulfonyl fluoride, pH 7.5) for 20 min on ice, followed by centrifugation at 13 000 g for 10 min at 4°C. After equal division, whole-cell lysates were incubated with 30 µl Protein G Sepharose 4 Fast Flow (GE Healthcare) and 1 µg monoclonal antibody or mouse IgG at 4°C for 3 h. The beads were collected by centrifugation and then washed gently three times with 1 ml lysis buffer containing 500 mM NaCl. Bound proteins were eluted by boiling in 2× SDS loading buffer and subjected to Western blotting. For endogenous co-immunoprecipitation analysis, Huh7.5.1 cells infected with JFH1 for 72 h and con1 replicon cells were directly lysed after washing with PBS.

For western blotting, samples were separated by SDS-PAGE, transferred to PVDF (Bio-Rad) and blocked with 5% skimmed milk in TBS. Blots were probed with different primary antibodies, followed by a secondary antibody conjugated to HRP. Protein bands were visualized with ECL Plus chemiluminescence reagent (Pierce).

## Results

### HCV NS5A Protein Interacts and Colocalizes with GPS2

**Figure 1:**
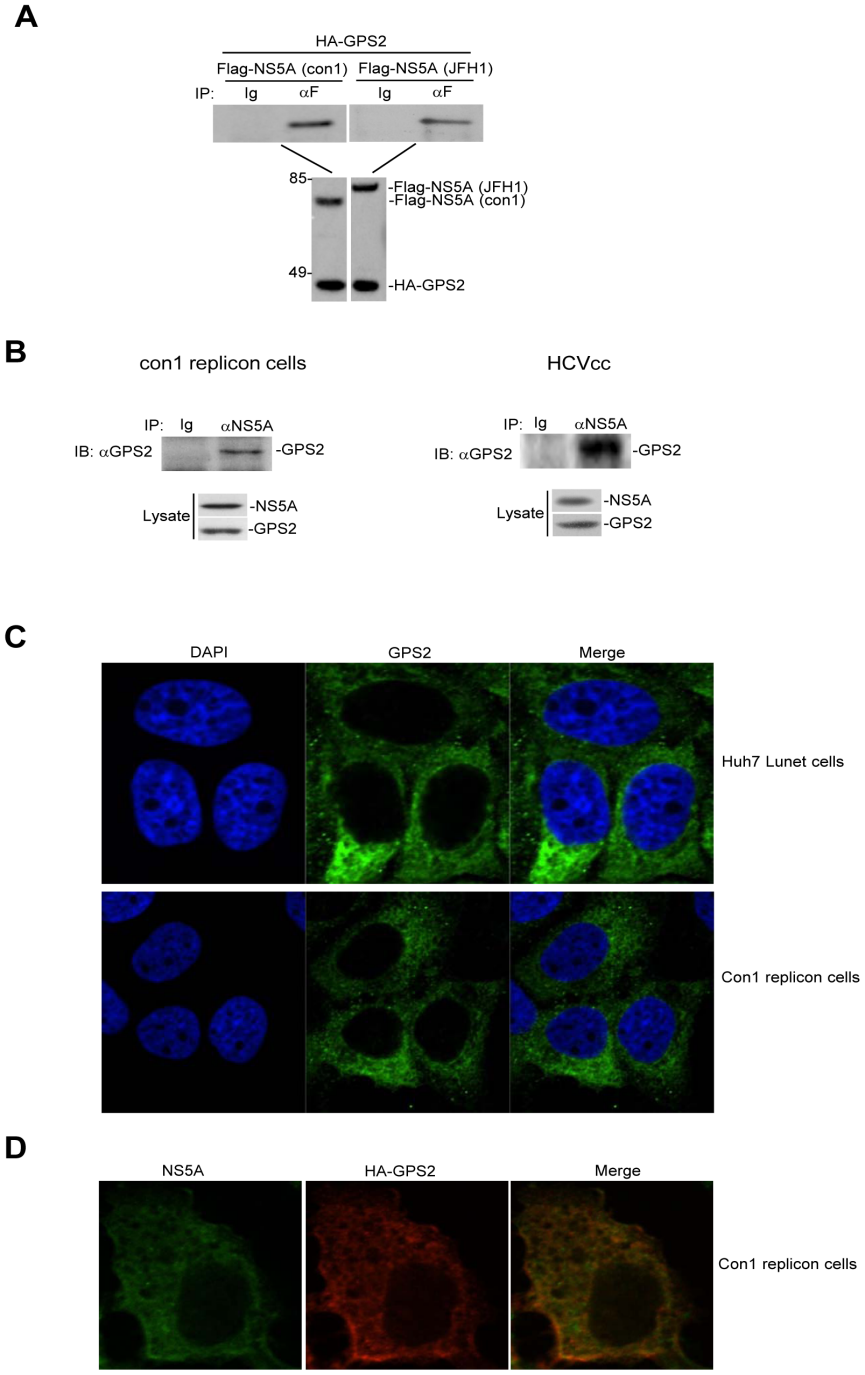
GPS2 interacts and colocalizes with NS5A. (A) GPS2 interacts with NS5A in transfected cells. 293T cells (2×10^6^) were transfected with the indicated plasmids (6 µg each). Cell lysates were immunoprecipitated with anti-Flag (F) antibody or control mouse IgG (Ig). The immunoprecipitates were analyzed by Western blots with anti-HA antibody (upper panel). The expression levels of the transfected proteins were analyzed by Western blots with anti-HA or anti-Flag antibodies (lower panel). (B) GPS2 associates with NS5A under physiological conditions. Con1 replicon cells (1×10^7^) and Huh7.5.1 cells (2×10^6^) infected with JFH1 or left uninfected for 72 h were harvested. Cell lysates were immunoprecipitated with anti-NS5A antibody or control mouse IgG (Ig). The immunoprecipitates were analyzed by Western blots with anti-GPS2 antibody (upper panel). The expression of individual protein was analyzed by Western blots with the indicated antibodies (lower panel). (C) GPS2 mainly localizes in the cytoplasm in Huh7 Lunet cells and Con1 replicon cells. Huh7 Lunet cells and Con1 replicon cells were stained with antibody to GPS2. Alexa Fluor 488-conjugated anti-mouse secondary antibody were used to detect GPS2 (green). Cells were stained with DAPI to visualize the nuclei (blue). (D) GPS2 colocalizes with NS5A in Con1 cells. Con1 cells were transfected with HA-GPS2 and stained with antibodies to HA and NS5A 24 h later. Alexa Fluor 488-conjugated anti-mouse secondary antibody and Alexa Fluor 555-conjugated anti-rabbit secondary antibody were used to detect NS5A (green) and HA-GPS2 (red), respectively.

GPS2 was identified to interact with HCV NS5A in the Y2H screens of human cDNA library recently [1]. To confirm this interaction in mammalian cells, we first examined the protein binding from GPS2 and NS5A transiently expressed cells by co-immunoprecipitation assay. HEK293T cells were cotransfected with a plasmid coding HA-GPS2 and a plasmid coding Flag tagged NS5A from either HCV genotype 1b or genotype 2a. As shown in Fig. 1A, NS5A derived from both genotypes interacted with GPS2. To further demonstrate protein interplay between GPS2 and NS5A in a more authentic system, HCV Con1 replicon cells and HCVcc infected Huh7.5.1 cells were immunoprecipitated with anti-NS5A antibody and bound proteins were then immunoblotted with anti-GPS2 antibody. Consistent with the data shown in Fig. 1A, GPS2 co-immunoprecipitated with NS5A of both the 1b and 2a genotypes in the context of HCV RNA replication (Fig. 1B). These results indicate that GPS2 specifically interacts with NS5A of both genotypes. GPS2 has been previously reported to predominantly localize to the nucleus [24], [25], while some study indicates ubiquitous cellular localization of endogenous GPS2 [21]. To investigate the subcellular localization of GPS2 in hepatoma cell lines, confocal immunofluorescence studies were performed in Huh7 Lunet cells and Con1 replicon cells. As shown in Figure 1C, GPS2 was mainly localized in the cytosol in both cell lines. To determine the relationship between subcellular localization of GPS2 and NS5A, the expression of GPS2 and NS5A in Con1 replicon cells transfected with HA-GPS2 was examined by immunofluorescence analyses. As shown in Fig. 1D, GPS2 colocalized with NS5A. These results further suggest that GPS2 interacts with NS5A in cells replicating HCV RNA.

### NS5A Interacts with Coiled-coil Domain of GPS2 through Domain I

**Figure 2:**
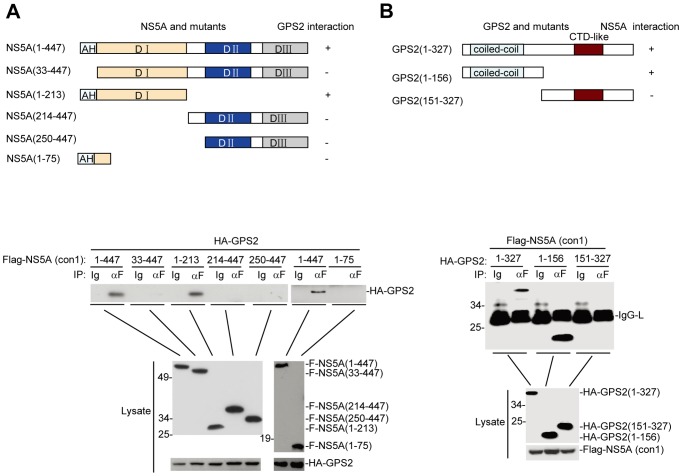
GPS2 interacts with domain I of NS5A through coiled-coil domain. (A) A schematic presentation of full-length NS5A, and their mutants and their interaction with GPS2. (B) A schematic presentation of full-length GPS2 and their mutants and their interaction with NS5A. 293T cells (2×10^6^) were transfected with the indicated expression plasmids (6 µg each). Cell lysates were immunoprecipitated with anti-Flag (F) antibody or control mouse lgG (Ig). The immunoprecipitates were analyzed by Western blots with anti-HA antibody (upper panel). The expression levels of the transfected proteins were analyzed by Western blots with anti-Flag or anti-HA antibody (Lower panel).

Since NS5A specifically interacts with GPS2, we tried to determine the regions of NS5A and GPS2 responsible for their interaction. First we determined the region in NS5A that is responsible for GPS2 binding. We constructed various truncation mutants of NS5A as previously reported (Fig. 2A) and the binding domain was determined by co-immunoprecipitation assay [26]. Fig. 2A showed that GPS2 interacted with NS5A mutant harboring domain I but not with mutant harboring domain II or III, indicating that GPS2 interacted with domain I of NS5A. It is noteworthy that the N-terminal amphipathic helix of NS5A that serves as a membrane anchor was also required for the interaction with GPS2, as NS5A mutant lacking this helix lose its ability to bind GPS2 (Fig. 2A) [27]. To test whether the N-terminal anchoring region alone binds to GPS2 or not, we constructed a NS5A mutant (1–75) which contains the N-terminal anchoring region and examined its binding to GPS2. The results (Fig. 2A) showed that NS5A (1–75) can’t bind to GPS2, which indicated the N-terminal anchoring region alone can’t bind to GPS2.

Then the interaction of NS5A with various truncation mutants of GPS2 (Fig. 2B) was determined as described above. As shown in Fig. 2B, NS5A interacted with GPS2 mutant encompassing aa 1–156, representing the coiled-coil domain, but not with aa 151–327 mutant of GPS2, indicating coiled-coil domain of GPS2 is responsible for NS5A binding.

### GPS2 is Required for Efficient HCV Replication

**Figure 3:**
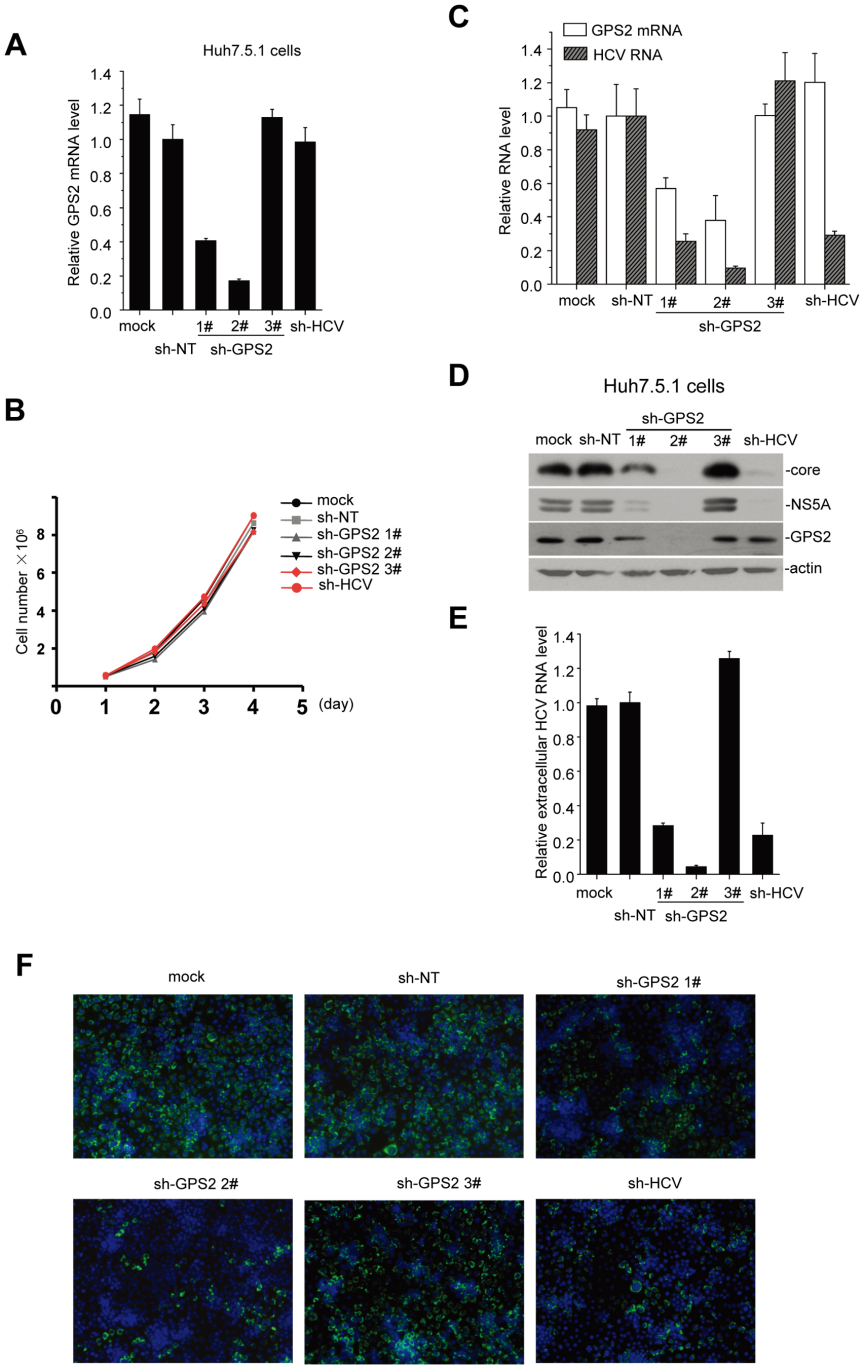
GPS2 is essential for HCV RNA replication. (A) Huh7.5.1 cells were transduced with the indicated short hairpin RNA (shRNA)-expression retroviral constructs or left untreated. GPS2 mRNA levels were determined by real-time qRT-PCR in these cells. The amount of GPS2 mRNA to that of glyceraldehyde-3-phosphate dehydrogenase (GAPDH) were calculated and graphed. (B) All cell lines were seeded respectively in 6-well plates with the same number and cell numbers were calculated at the indicated time point and graphed. (C) Huh7.5.1 cells expressing shRNAs were infected with JFH1 at a multiplicity of infection (MOI) of 0.5. At 72 h postinfection, the levels of intracellular HCV RNA and GPS2 mRNA were analyzed by qPCR. (D) Total cell lysates harvested from these cells were immunoblotted with the indicated antibodies. (E) Extracellular RNAs isolated from the culture supernatant were quantified by qPCR. Experiments were carried out in duplicate. Error bars indicate standard deviations. (F) Cell culture supernatants were used to infect naive Huh7.5.1 cells plated in 96-well plate. At 72 h postinfection, cells were stained for HCV core with anti-core antibody and stained with the DAPI before confocal microscopy.

Since GPS2 associates with HCV NS5A protein, we hypothesize that GPS2 may play a role in HCV replication. To investigate whether GPS2 is involved in HCV replication, we suppressed GPS2 expression in Huh7.5.1 cells using a retrovirus-based expression system allowing the transcription of short hairpin RNA (shRNA). GPS2 mRNA levels of knockdown cells were evaluated by qRT-PCR assay (Fig. 3A). GPS2 levels in sh-GPS2-transduced cells (1# and 2#) were specifically reduced compared to the non-target sh-NT control cells and sh-HCV cells. Here, sh-GPS2 3#, which had no impact on GPS2 expression, was also set as a control in the following experiments. Importantly, all these shRNA delivery did not affect cell viabilities (Fig. 3B).

We next tested the GPS2 knockdown cell lines for their capacities of supporting HCV RNA replication. Transduced Huh7.5.1 cells were infected with JFH1 virus at an MOI of 0.5. Intracellular HCV RNA level as well as the expression levels of HCV core and NS5A proteins was examined three days post-infection. As shown in Fig. 3C, intracellular HCV RNA level was profoundly reduced in GPS2 knockdown cells, nearly by 90% reduction in sh-GPS2 2# cells, compared to those of sh-NT-transduced cells. At the same time, HCV RNA level was decreased by 80% in sh-HCV cells as expected. Similar results were observed with another shRNA depleting GPS2 (sh-GPS2 1#), excluding an off-target effect of sh-GPS2 (Fig. 3C). While sh-GPS2 3# had no effect on GPS2 expression level, it also did not show any impact on HCV RNA replication. Accordingly, the expression of HCV core and NS5A proteins were also severely decreased in GPS2 knockdown cells (Fig. 3D). The fact that suppression of viral RNA replication and viral protein expression closely coincided with GPS2 expression level, suggested that GPS2 is particularly required for efficient HCV replication.

Given that the HCV replication level is lower in shGPS2 cells, we further measured viral release and viral infectivity in the culture supernatants. Results showed that HCV RNA level and viral titers of supernatant were also decreased accordingly in GPS2 knockdown cells (Fig. 3E and F). Taken together, these results indicate that GPS2 significantly contributes to HCV replication.

### Transduction of GPS2 Rescues HCV Replication in GPS2 Knockdown Cells

**Figure 4:**
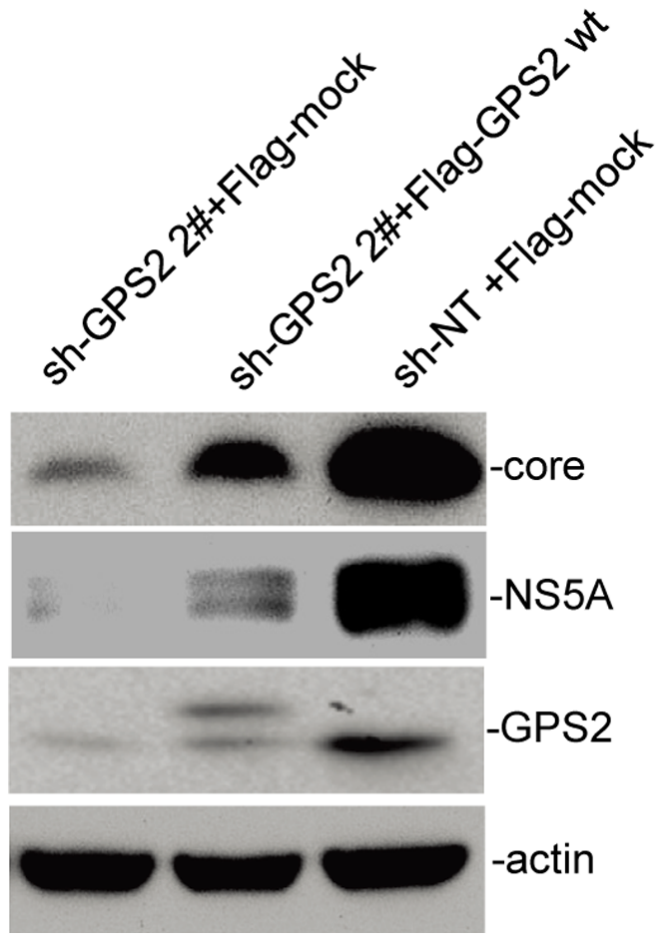
Transduction of GPS2 rescues HCV replication in GPS2 knockdown cells. Huh7.5.1 cells with stable knockdown of GPS2 expression (sh-GPS2) were stably transduced with shRNA-resistant Flag-tagged GPS2 expression construct. For comparison, the GPS2 knockdown cells and Huh7.5.1 cells expressing a non-target shRNA (sh-NT) were transduced with the empty plasmid in parallel. Cell lines were infected with JFH1 with a MOI of 0.5. Virus replication was determined by Western blots 72 h after infection.

For rigorous exclusion of off-target effects and to further demonstrate the significance of GPS2 in HCV replication, rescue cell lines were established through stable expression of shRNA-resistant Flag-tagged GPS2 in GPS2 knockdown cells. Flag-tagged GPS2 and empty control were packaged in retrovirus which was used to infect stable knockdown cells. As the target sequence of sh-GPS2 2# is located in the 3′UTR of GPS2 mRNA, the transduced Flag-tagged GPS2 was resistant to the knockdown regulation. Upon infection of the GPS2 rescue cell line with JFH1, the level of HCV replication was comparable to that in the sh-NT control cells as judged by the amounts of core and NS5A proteins (Fig. 4). However, no rescue was obtained in cells stably transduced with an empty control vector. These findings strongly suggest that GPS2 is essential for HCV RNA replication.

### GPS2 is Required for the Interaction between VAP-A and NS5A

**Figure 5:**
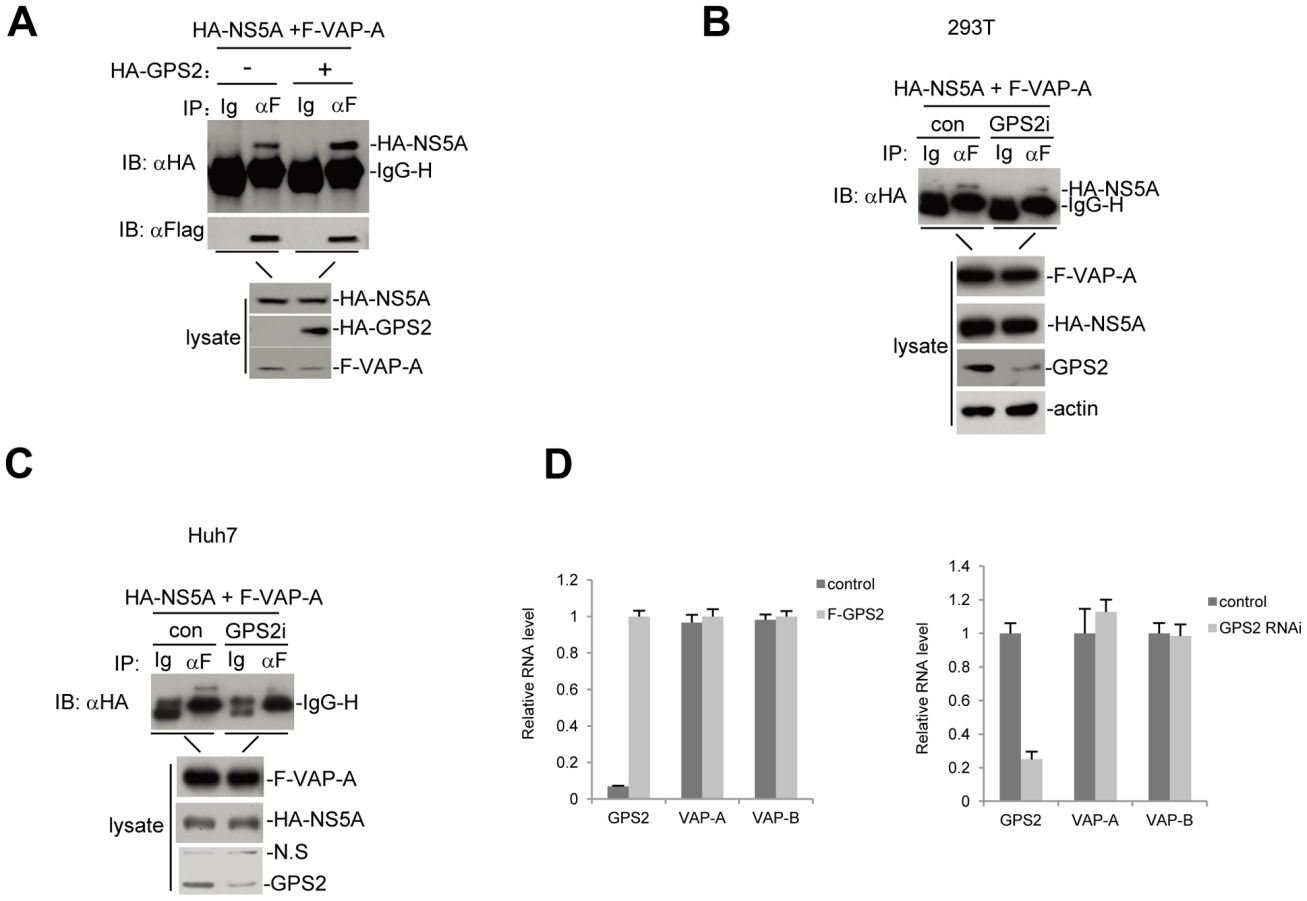
GPS2 is indispensable for the interaction between NS5A and VAP-A. (A) Overexpression of GPS2 enhances the interaction between NS5A and VAP-A. 293T cells (2×10^6^) were transfected with the indicated expression plasmids (6 µg each). Cell lysates were immunoprecipitated with anti-Flag (F) antibody or control mouse lgG (Ig). The immunoprecipitates were analyzed by Western blots with anti-HA antibody (upper panel). The expression levels of transfected proteins were analyzed by Western blots with anti-Flag or anti-HA antibody (Lower panel). (B) Knockdown of GPS2 disrupts the interaction between NS5A and VAP-A in 293T cells. 293T cells (2×10^6^) were transfected with the indicated plasmids. Cell lysates were immunoprecipitated with anti-Flag (F) antibody or control mouse lgG (Ig). The immunoprecipitates were analyzed by Western blots with anti-HA antibody, while the expression levels of proteins in cell lysates were analyzed by Western blots with indicated antibody. (C) Knockdown of GPS2 disrupts the interaction between NS5A and VAP-A in hepatoma cells. sh-GPS2 or sh-NT transduced stable Huh7 cell lines were transfected with the indicated plasmids. Cell lysates were immunoprecipitated with anti-Flag (F) antibody or control mouse lgG (Ig). The immunoprecipitates were analyzed by Western blots with anti-HA antibody, while the expression levels of proteins in cell lysates were analyzed by Western blots with indicated antibody. (D) Neither overexpression nor knockdown of GPS2 influences VAP-A/B expression. Huh7 cells were transiently transfected with Flag-GPS2 or control vector. VAP-A/B mRNA levels were determined by qRT-PCR 48 h later in these cells. The amount of VAP-A/B mRNA to that of GAPDH were calculated and graphed. In another assay, VAP-A/B mRNA levels in Huh7 cells with stable knockdown of GPS2 expression and control cells were determined by real-time qRT-PCR.

To further investigate the contribution of GPS2-NA5A interaction to HCV RNA replication, we examined whether GPS2 affects interactions among HCV NS5A and other host cellular factors that have been reported to be involved in HCV replication, such as VAP-A [13]. HEK293T cells were cotransfected with HA-tagged NS5A and Flag-tagged VAP-A expression plasmids, in the presence or absence of HA-GPS2. Protein binding were examined by coimmunoprecipitation and Western blotting at 24 h after transfection. As shown in Fig. 5A, HA-NS5A was coimmunoprecipitated by Flag-VAP-A in the control group without GPS2 overexpression, which is consistent with previous results [13]. However, in the presence of GPS2, the level of coimmunoprecipitated HA-NS5A was significantly increased, while the total expression levels of Flag-VAP-A and HA-NS5A were equivalent to those of the control group (Fig. 5A).

We further determined the impact of GPS2 on NS5A association with VAP-A in GPS2 knockdown cells. First, we transiently cotransfected 293T cells with HA-NS5A and Flag-VAP-A and sh-GPS2 2# or sh-NT control plasmids, cells were harvested 48 h later and co-immunoprecipitation experiments were performed. As shown in Fig. 5B, NS5A association with VAP-A was obviously reduced in sh-GPS2 cells compared to sh-NT cells. Similar result was observed in Huh7 hepatoma cells (Fig. 5C). When we transfected HA-NS5A and Flag-VAP-A into sh-GPS2 or sh-NT transduced Huh7 cells, association between NS5A and VAP-A was severely impaired in sh-GPS2 cells. Considering the role of GPS2 in transcriptional regulation through interaction with a series of transcriptional regulators, we examined whether GSP2 affects expression of VAP-A and VAP-B. In Huh7 cells, neither overexpression nor knockdown of GPS2 affected VAP-A/B expression (Fig. 5D). These results suggest that GPS2 modulates interaction between NS5A and VAP-A, instead of regulating VAP-A/B expression. Together, our results indicate that GPS2 is critical for efficient association between NS5A and VAP-A, and is also obligatory for robust HCV replication.

## Discussion

In the study to generate a comprehensive interactome map between HCV and cellular proteins, de Chassey B *et al*. screened human cDNA library by a yeast two-hybrid system using full-length HCV mature proteins or discrete domains as baits and identified GPS2 as a candidate binding protein to NS5A [1]. In this article, we first demonstrate the interaction between GPS2 and NS5A by co-immunoprecipitation assay. Then we showed that knockdown of GPS2 significantly reduces extracellular HCV RNA level as well as intracellular HCV replication. These results suggest that host factor GPS2 is required for HCV replication.

GPS2 is a 37-kDa protein ubiquitously expressed in human tissues, including liver. Several studies have reported that GPS2 participates in transcriptional regulation through acting as a transcriptional co-activator or co-repressor [19], [20], [28], [29]. In concert with its nuclear roles, the pivotal role of GPS2 functioning at the level of the plasma membrane to control the TNF-α signaling pathway was uncovered recently [21]. Here we demonstrate that GPS2 interacted with HCV NS5A in mammalian overexpression system as well as in the HCV subgenomic replion cells and HCV infected cells. Domain mapping experiments indicate coiled-coil domain of GPS2 and domain I of NS5A are responsible for their interaction, and the N-terminal α-helix of NS5A is also strictly needed. But the N-terminal helix region alone can’t bind to GPS2. It has been previously reported that this helix of NS5A mediated membrane association and deletion of the amino-terminal 44 aa resulted in a nuclear translocation of the NS5A protein [27]. Interestingly, another two host cellular factors, VAP-A and VAP-B, have been reported to interact with HCV NS5A through coiled-coil domain [13], [16]. GPS2 has been previously reported to predominantly reside in the nucleus, while some study indicates ubiquitous cellular localization of endogenous GPS2 [21], [24], [25]. However, in our study we found that GPS2 mainly localizes in the cytoplasm in Huh7 Lunet cells and Con1 replicon cells and colocalizes with NS5A. These results suggest the localization of GPS2 may vary greatly in different cell types, which allowing it to exert various functions.

NS5A plays key roles in multiple aspects of the virus life cycle. As well as involvement in genome replication (mainly mapping to domain I and part of domain II), the protein has recently been shown to be important in the assembly of infectious virus particles through interacting with core around lipid droplet (LD) [30], [31]. Domain I of NS5A has also been reported to mediate interactions with other host factors, such as PI4KIIIα [32]. The overlapping of binding region within NS5A suggests interplay between different cellular pathways involved in HCV replication. Knockdown of GPS2 led to impaired HCV RNA replication, which can be restored by the expression of shRNA-resistant GPS2. These results suggest that the impairment of HCV RNA replication induced by the knockdown of GPS2 was not due to an off-target effect of shRNA against GPS2.

To elucidate how GPS2 and its interaction with NS5A influence HCV replication, we wondered whether GP2 competed or coordinated with other cellular factors to interact with HCV NS5A. We demonstrated that GPS2 facilitates the association of NS5A with VAP-A, which is essential for efficient viral RNA replication [15]. Although the exact mechanism of how VAP-A enhances HCV replication is unknown, VAP-A is thought to be involved in the formation of HCV replication complex [15]. Furthermore, knockdown of GPS2 obviously impaired NS5A association with VAP-A in different cell types, which may explain the role of GPS2 in HCV replication. Importantly, neither overexpression nor knockdown of GPS2 affects VAP-A/B expression, indicating the effects of GPS2 on interaction between NS5A and VAP-A are not due to its transcriptional regulation of VAP-A expression, Another study has demonstrated that the ability of binding VAP-A is modulated by the phosphorylation state of NS5A [33]. GPS2 association with NS5A may regulate its phosphorylation state to facilitate the interaction of NS5A with VAP-A, which requires further investigation. Also, viperin, an interferon-stimulated gene, is reported to interact with VAP-A and inhibit HCV replication through interfering its interaction with NS5A [34], [35]. These results indicate modulation of NS5A association with VAP-A may be an effective way to regulate HCV replication. Based on the former studies and our research, we hypothesize that after the HCV polypeptide is translated and cleaved, HCV nonstructural proteins are recruited to lipid raft by the intrinsic lipid raft-anchoring property or cofactors. GPS2 is then recruited by NS5A and binds to its N-terminal α-helix, which facilitates the interaction between NS5A and VAP-A. VAP-A can also binds to NS5B. Interactions of VAP-A with both NS5A and NS5B benefit the formation of HCV replication complex on lipid raft. But how GPS2 affects VAP-A and NS5A interaction needs further investigation.

In this study, we demonstrate cellular factor GPS2 as a binding partner of NS5A and determine that GPS2 facilitates the association of NS5A with VAP-A, and is required for HCV replication. This work adds to our understanding of interplay between HCV and host cells. Further studies to elucidate the precise roles of GPS2 and its interaction with NS5A in HCV replication should provide valuable insights into viral host interaction and development of novel therapeutics for chronic hepatitis C.
